# Optimal sway motion reduction in forestry cranes

**DOI:** 10.3389/frobt.2024.1417741

**Published:** 2024-08-15

**Authors:** Elham Kowsari, Reza Ghabcheloo

**Affiliations:** Faculty of Engineering and Natural Sciences, Tampere University, Tampere, Finland

**Keywords:** sway damping, optimal control, forestry machinery automation, forwarder, feedforward (FF) control

## Abstract

**Introduction:**

The paper introduces a novel optimal feedforward controller for Hydraulic manipulators equipped with a passive grapple, addressing the issue of sway during and after movement. The controller is specifically applied to a forwarder machine used in forestry for log-loading tasks.

**Methods:**

The controller is designed for smooth operation, low computational demands, and efficient sway damping. Customizable parameters allow adjustments to suit operator preferences. The implementation was carried out using the Amesim model of a forwarder.

**Results:**

Simulation results indicate a significant reduction in sway motions, averaging a decrease of more than 60%. This performance was achieved without the need for additional sway-detection sensors, which simplifies the system design and reduces costs.

**Discussion:**

The proposed method demonstrates versatility and broad applicability, offering a new framework for anti-sway controllers in various fields such as construction cranes, forestry vehicles, aerial drones, and other robotic manipulators with passive end-effectors. This adaptability could lead to significant advances in safety and efficiency.

## 1 Introduction

**FIGURE 1:**
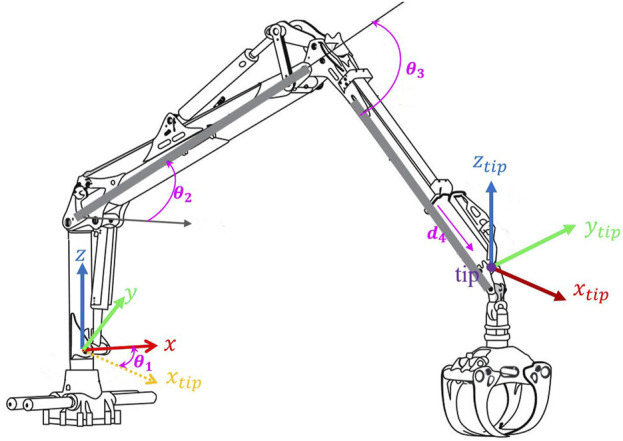
Forwarder Crane and tip position in cylindrical coordinate system according to Denavite-Hartenberg [modified from (Hera and Morales, 2015)].

The forestry industry is vital to many countries’ economies. However, unlike other industries like mining and agriculture, it is behind in using robots and innovative technology. There is a growing need to make forestry machines more intelligent and autonomous, mainly because there are not enough trained people to operate them. Operating these machines safely and effectively requires much training because the job is physically and mentally demanding (Jebellat and Sharf, 2023). Like moving robots, forestry machines have mobile and crane parts with a unique tool at the end-effector. Operating these giant cranes is very complicated. Studies show that operators spend most of their time (over 80%) controlling the crane (Dvořák et al., 2008). Also, many crane accidents (73%) happen because of human mistakes (Brkić et al., 2015). So, adding some automation to these machines could help make the operator’s job easier and make things safer in the forestry industry (Dvořák et al., 2008). Research efforts have been increasingly directed toward enhancing the autonomy of various forestry machinery, including harvesters and forwarders. Among these, the forwarder (shown in Figure 1) plays a pivotal role in the forestry operation cycle by collecting cut logs from the forest and transporting them to roadside depots for further processing or transport. The forwarder’s key components include an operator’s cabin, a storage basket or trailer for logs, a crane (or boom) with four actively controlled degrees of freedom (DOF), and a grapple at the crane’s tip connected via two passive DOF. These passive joints are designed to maintain the grapple’s vertical orientation, aiding in efficiently handling logs. Nonetheless, the inability to actively control these joints leads to unwanted sway or oscillation of the grapple during and after crane operations (Ayoub et al., 2023). The swaying motion, worsened by the heavy weight of the grapple and the additional burden of logs, presents serious hazards. This movement can unintentionally cause the grapple to hit surrounding trees or even the operator’s cabin, harming the forest, equipment, and possibly the operator. Consequently, managing the grapple’s sway adds a considerable challenge for operators, impacting their workload and safety. Moreover, the residual sway- the grapple’s continuing oscillation after crane movements have ceased— directly impacts operational efficiency. Operators often must pause operations, waiting for the sway to subside before safely continuing, affecting the overall productivity of forestry operations. This issue is further compounded when considering integrating autonomy-enhancing technologies, such as crane-mounted cameras, for environmental perception, where steady positioning is crucial for accurate data capture and analysis. Addressing the sway of the grapple promises to alleviate the operational burden on human operators and significantly improves the safety and efficiency of forestry machinery operations, paving the way for more sophisticated autonomous capabilities (Qiang et al., 2021). Developing and implementing anti-sway techniques are crucial across various industries, not just within the forestry industry (Sadr et al., 2014; Reis et al., 2023). The stabilization of sway motion in suspended payloads is essential in numerous applications, including the operation of construction cranes (such as tower and gantry cranes), the management of payloads by quad-rotors (Fielding and Nahon, 2019), and the manipulation of hanging loads by robotic arms. To address the challenges of sway, researchers have innovated and applied various anti-sway methodologies at both the trajectory planning and control system design stages. Among these strategies, input shaping as an advanced feedforward control has been widely adopted across several applications executing rest-to-rest maneuvers to manage residual vibrations effectively (Cole, 2011). This technique, proven its efficiency through deployment in diverse practical systems such as cranes, telescopic handlers, industrial robots, and coordinate measuring machines, involves using a carefully designed sequence of impulses (known as the input shaper) in convolution with a specific system command. This combination precisely tailors the system’s input to achieve the desired motion without sway. However, the effectiveness of this method in reducing the sway motion relies on precisely calculating the impulses’ amplitudes and timing, which depend on the system’s parameters. Incorrect estimates in these areas can result in residual vibrations compromising system performance. Additionally, the traditional input shaping approach does not address vibrations caused by external disturbances (Pai, 2012) and is only suitable for rest-to-rest situations. To further enhance the system’s resilience against uncertainties in its model parameters, it’s possible to integrate extra impulses into this sequence, albeit at the expense of extended operation times. Additionally, innovative adaptive input shaping methods have been formulated (Solatges et al., 2017), aiming to bolster robustness while concurrently seeking to reduce the length of the impulse sequence, thereby offering a refined balance between system stability and efficiency (ur Rehman et al., 2022).

In this context, feedback control methods are effective, although they are often associated with increased computational requirements (Kalmari et al., 2014; Yousefi et al., 2022). However, this method sometimes results in trajectories that need more smoothness, rendering them impractical for real-world applications. Even though closed-loop control usually does a better job at handling external disturbances and unexpected changes, it has its downsides because it needs special equipment and the creation of a controller.

Beyond open and closed-loop controllers, researchers have explored using customized motion planners to mitigate sway motion. For example, in (Jebellat and Sharf, 2023), dynamic programming generates a trajectory for the forwarder and reduces sway motions. This motion planner is designed in the joint space, and the state vector includes all joint angles, velocities, and accelerations. Due to the high dimension of the system considered, this approach results in significant computational complexity.

Each method has pros and cons, showing that it’s important to think carefully about the needs and limits of the specific situation. The continued improvement of anti-sway technology emphasizes how crucial precision, efficiency, and safety are in automating systems where controlling hanging loads is very important. As this area evolves, refining these techniques and finding new solutions are crucial for enhancing how automated systems safely and effectively handle sway. In this paper, we have developed an innovative control mechanism that significantly reduces the sway in forestry machines by over 60%, eliminating the need for additional, complex sensors that feedback controllers require to monitor sway motion. This advancement streamlines the design and substantially cuts costs, offering a practical and efficient solution for sway control. Our controller operates highly efficiently and demands minimal computational resources, ensuring smooth operation. It demonstrates high adaptability and reliability, even in scenarios with initial sway, unlike other input shaping and motion planners suited only for rest-to-rest conditions. This achievement underscores our significant contribution to enhancing operational safety and cost efficiency in forestry machinery through advanced control technologies. A high accuracy and fast control system will require both feedback and feedforward controllers working together. Feedforward controllers provide fast response to control commands and brings the system trajectories close to desired ones, while feedback controllers remove the effect of unmodeled dynamics and disturbances, but require sensor measurements. In this paper, we focus on the feed forward controller parameter tuning. In the rest of the paper, we delve into the details: Section 2 explains the forwarder and grapple model and derives the mathematical model for sway motion. Section 3 introduces our new control strategy to reduce sway motion and optimizations for better performance. Section 4 presents simulation results, demonstrating the effectiveness of our proposed controller on the Forwarder model. Finally, we conclude by discussing the results and highlighting the significance of our advancements in improving machine safety and efficiency.

## 2 Model of forwarder and grapple

The kinematic structure of the crane’s boom is characterized by four degrees of freedom (DOF): three of them are rotary, one is prismatic. However the linear hydraulic actuators create closed loop kinematic chains, but they are not considered in this study, since the control inputs are directly the joint angle speeds, the mechanism in our study is modeled as an open kinematic chain, as shown in Figure 1. Typically, the main goal involves manipulating the boom’s tip within a three-dimensional space, which introduces an additional degree of freedom beyond what is strictly necessary, rendering the crane a redundant manipulator (Liu et al., 2022). This redundancy brings both benefits and drawbacks. Four degrees of freedom enhance the boom’s reach, extending to greater distances and offering increased operational flexibility (Zhou et al., 2019). However, the complexity of controlling such a manipulator escalates due to the lack of a direct, one-to-one correlation between the position of the boom’s tip and the joint configurations. Consequently, devising a control strategy for a redundant manipulator demands a more intricate approach. Before delving into the grapple model details, let’s define the four main boom motions on the forwarder: a) *Extension* refers to the crane’s ability to lengthen or extend its boom. b) *Near and Far*: “Near” indicates the direction closer to the crane’s base, while “far” denotes the direction farther away from the base. c) *Slewing*, slewing involves the horizontal movement of the crane. d) *Lift* involves raising or lowering the load using the crane, allowing the operator to pick up a load from the ground and transport it. Our experiments highlight that slewing and near-far motions significantly influence sway movement. Slewing induces sway motions in both perpendicular and parallel directions, while near-far motion causes sway motions primarily in the parallel direction with the boom tip. In the following, we have determined nonlinear mapping that considers slewing and tip commands for controlling sway over both slewing and near-far motions.

The forwarder crane features two passive joints that link the end effector (EE) — the grapple — to the boom. The sway of the EE is primarily determined by the movement of the boom tip, which is, in turn, significantly influenced by the activity of the joints preceding it.

**FIGURE 2:**
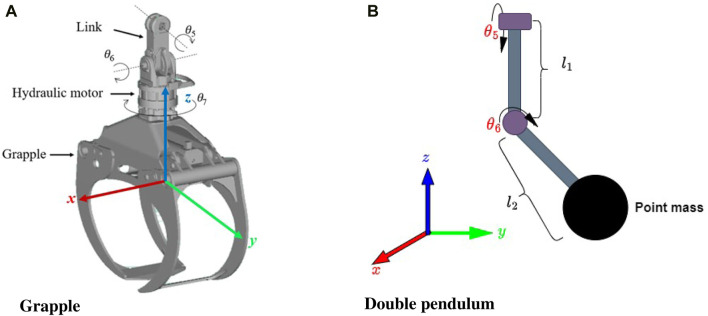
Grapple and the related coordinate frame **(A)** The grapple system is modeled as a pendulum system and a point mass **(B)**.

The grapple is connected to the boom by two suspended links, and any rapid motion commands from the operator cause the grapple to sway, making it more challenging to maneuver the boom. The grapple consists of three rotational joints, namely $\theta_{5}$ perpendicular, $\theta_{6}$ parallel motion with boom, and $\theta_{7}$ turning of the grapple by hydraulic motor as illustrated in Figure 2. The upper joint $\theta_{5}$ connects the entire grapple to the tip of the crane. The rotation around these $\theta_{5}$ and $\theta_{6}$ joints is free and cannot be directly manipulated and controlled.

In Figure 1, the position of the boom’s tip in Cartesian coordinates, as determined by the joint angles, is calculated using Denavit-Hartenberg notation. The pose, which includes both the position and orientation of an individual link i relative to the preceding link, is defined by a homogeneous transformation matrix.

$$T_{i}^{\;i-1}=\left(\begin{array}{cccc} \cos\left(\theta_{i}\right) & -\sin\left(\theta_{i}\right) & 0 & a_{i}\\ \sin\left(\theta_{i}\right)\cos\left(\alpha_{i}\right) & \cos\left(\theta_{i}\right)\cos\left(\alpha_{i}\right) & -\sin\left(\alpha_{i}\right) & -\sin\left(\alpha_{i}\right)d_{i}\\ \sin\left(\theta_{i}\right)\sin\left(\alpha_{i}\right) & \cos\left(\theta_{i}\right)\sin\left(\alpha_{i}\right) & \cos\left(\alpha_{i}\right) & \cos\left(\alpha_{i}\right)d_{i}\\ 0 & 0 & 0 & 1 \end{array}\right)$$ (1)

where $a_{i}$ , $d_{i}$ , $\alpha_{i}$ , and $\theta_{i}$ are the parameters describing the structure and current state of the boom, For more details regarding these parameters, please refer to Kalmari et al. (2014). The total transformation $T_{\mathrm{tip}}$ from the base to the tip can be obtained by multiplying all the individual transformation matrices.

$$T_{\text{tip}}=T_{1}^{0}\left(\theta_{1}\right)T_{2}^{1}\left(\theta_{2}\right)T_{3}^{2}\left(\theta_{3}\right)T_{4}^{3}\left(d_{4}\right),$$ (2)

Then the position of the tip is:

$$tip_{\text{p}}=\left(\begin{array}{c} \cos\left(\theta_{1}\right)\left(a_{2}-d_{4}\sin\left(\theta_{2}+\theta_{3}\right)+a_{4}\cos\left(\theta_{2}+\theta_{3}\right)+a_{3}\cos\left(\theta_{2}\right)\right)\\ \sin\left(\theta_{1}\right)\left(a_{2}-d_{4}\sin\left(\theta_{2}+\theta_{3}\right)+a_{4}\cos\left(\theta_{2}+\theta_{3}\right)+a_{3}\cos\left(\theta_{2}\right)\right)\\ d_{1}+d_{4}\cos\left(\theta_{2}+\theta_{3}\right)+a_{4}\sin\left(\theta_{2}+\theta_{3}\right)+a_{3}\sin\left(\theta_{2}\right) \end{array}\right)$$ (3)

The controller’s objective is to maneuver the boom’s tip while reducing the sway motion of the connected grapple. This necessitates the development of a model that indicates the sway motions based on the tip commands $\left[\dot{x},{\dot{\theta }}_{1},\dot{z}\right]$ , where $\dot{x},\;{\dot{\theta }}_{1}$ and $\dot{z}$ are tip velocity in direction x , slewing angular velocity and tip velocity in direction z , respectively. The grapple model can be simplified by making some assumptions; one assumption is that its mass is concentrated at a single point at the end of the two link parts. Therefore, the grapple model can be considered a double pendulum, as shown in Figure 2B. The double pendulum is a classic problem in dynamics, illustrating complex motion that can be chaotic under certain conditions. This simplification eliminates the consideration of rotational inertia in the dynamic equations but dramatically simplifies the model (Kalmari et al., 2013). Since the angular velocities of the grapple are low, the rotational inertia is negligible and can be disregarded. The analysis considers only gravity and the forces generated by the movement of the boom tip while disregarding external forces. Moreover, the influence of viscous friction on the joints is also considered. In Figure 2, $l_{1}$ represents the distance between the first and second joint, while $l_{2}$ corresponds to the distance from the second joint to the center of mass of the grapple. The third rotational degree of freedom $\theta_{7}$ is not currently of interest because the swaying motion occurs in the freely rotating joints. The dynamic equations for the sway motion can be derived by utilizing the Lagrangian method; for more details, please refer (Kalmari et al., 2013). The equations describing the sway dynamics of the grapple are derived as follows:

L=T−V (4)

where the Lagrangian (L) isidentified by the difference between the system’s kinetic energy (T) and potential energy (V) as shown in Eq. 4. For the sake of brevity, the formulations of T and V are described in the Appendix A. The torques influencing a specific joint ’i’ can be described as follows:

$$\tau_{i}=\frac{d}{dt}\frac{\partial L}{\partial \dot{\theta_{i}}}-\frac{\partial L}{\partial \theta_{i}}\;\;\;i=5,6$$ (5)

In this context, $\tau_{i}$ represents the torque at joint i , specifically the damping torque at the joint for the two passive joints connected to the grapple. This damping torque is modeled as viscous friction, characterized by a damping coefficient $c_{i}$ . The damping torques can be shown as Eq. 6.

$$\tau_{i}=-c_{i}{\dot{\theta }}_{i},\;\;i=5,6$$ (6)

Finally, the equations describing the sway dynamics of the grapple are derived as follows:

$$\begin{array}{cc} \dot{\theta_{5}}= & -\frac{1}{c_{5}}\left[\left(m_{1}l_{1}+m_{2}l_{2}\right)\left(cos\left(\theta_{5}\right)cos\left(\theta_{1}\right){\ddot{x}}_{\mathrm{tip}}+cos\left(\theta_{5}\right)sin\left(\theta_{1}\right){\ddot{y}}_{tip}\right.\right.\\ & \;\left.+sin\left(\theta_{5}\right)\left({\ddot{z}}_{\mathrm{tip}}+g\right)\right)+m_{1}l_{1}\left(l_{1}{\ddot{\theta }}_{5}-l_{1}sin\left(\theta_{5}\right)cos\left(\theta_{5}\right){\dot{\theta }}_{1}^{2}\right)\\ & \;+m_{2}l_{3}\left(l_{3}{\ddot{\theta }}_{5}-2l_{2}sin\left(\theta_{6}\right)\dot{\theta_{5}}\dot{\theta_{6}}-l_{2}cos\left(\theta_{5}\right)sin\left(\theta_{6}\right){\ddot{\theta }}_{1}\right.\\ & \;\left.\left.-l_{3}sin\left(\theta_{5}\right)cos\left(\theta_{5}\right){\dot{\theta }}_{1}^{2}-2l_{2}cos\left(\theta_{5}\right)cos\left(\theta_{6}\right){\dot{\theta }}_{1}{\dot{\theta }}_{6}\right)\right] \end{array}$$ (7)

$$\begin{array}{cc} \dot{\theta_{6}}= & -\frac{1}{c_{6}}\left[m_{2}l_{2}\left({\ddot{x}}_{tip}\left(-cos\left(\theta_{6}\right)sin\left(\theta_{1}\right)-sin\left(\theta_{5}\right)sin\left(\theta_{6}\right)cos\left(\theta_{1}\right)\right)\right.\right.\\ & \;+{\ddot{y}}_{tip}\left(cos\left(\theta_{6}\right)cos\left(\theta_{1}\right)-sin\left(\theta_{5}\right)sin\left(\theta_{6}\right)sin\left(\theta_{1}\right)\right)+l_{2}{\ddot{\theta }}_{6}\\ & \;+cos\left(\theta_{5}\right)sin\left(\theta_{6}\right)\left({\ddot{z}}_{tip}+g\right)+sin\left(\theta_{5}\right)\left(l_{1}cos\left(\theta_{6}\right)+l_{2}\right){\ddot{\theta }}_{1}\\ & \;+sin\left(\theta_{6}\right)\left(l_{1}-l_{3}cos^{2}\left(\theta_{5}\right)\right){\dot{\theta }}_{1}^{2}+l_{3}sin\left(\theta_{6}\right){\dot{\theta }}_{5}^{2}\\ & \;\left.+2l_{3}cos\left(\theta_{5}\right)cos\left(\theta_{6}\right)\dot{\theta_{1}}\dot{\theta_{5}}\right] \end{array}$$ (8)

where $l_{3}=l_{1}+l_{2}cos\left(\theta_{6}\right)$ , and the constant “ g ” denotes gravity, while “ $m_{1}$ ” and “ $m_{2}$ ” stand for the pendulum’s masses in Figure 2 and $c_{5}$ and $c_{6}$ are the viscous friction coefficients in the joint 5 and 6, respectively. ${\ddot{x}}_{\mathrm{tip}}$ , ${\ddot{y}}_{\mathrm{tip}}$ , and ${\ddot{z}}_{\mathrm{tip}}$ are the accelerations of the tip of the boom in base coordinate system which are calculating based on Eq. 3, respectively.

## 3 Proposed controller

The sway motion formula has been derived in the previous section. Now, this model must be delved deeper into to gather additional information. Eqs 7, 8 depict the sway motions based on the tip acceleration, expressed in the Cartesian base coordinate system $\left(x_{\mathrm{tip}},y_{\mathrm{tip}},z_{\mathrm{tip}}\right)$ (see Figure 1), since is operated in cylindrical coordinate system $\left(\theta_{1},x,z\right)$ presented in Figure 1. The controller is designed in the task space, Eqs 7, 8 are rewritten based on the cylindrical coordinate system.

$$\begin{array}{cc} {\ddot{x}}_{\mathrm{tip}} & =sin\left(\theta_{1}\right)\left(\ddot{x}-x{\dot{\theta }}_{1}\right)-cos\left(\theta_{1}\right)\left(x\ddot{\theta_{1}}+2\dot{x}\dot{\theta_{1}}\right)\\ {\ddot{y}}_{\mathrm{tip}} & =cos\left(\theta_{1}\right)\left(\ddot{x}-x{\dot{\theta }}_{1}\right)+sin\left(\theta_{1}\right)\left(x\ddot{\theta_{1}}+2\dot{x}\dot{\theta_{1}}\right)\\ {\ddot{z}}_{\mathrm{tip}} & =\ddot{z} \end{array}$$ (9)

By substituting Eq. 9 in Eqs 7, 8, the sway motion is expressed within the cylindrical coordinate system. Since the sway motion model is strictly nonlinear, analyzing it directly from the differential equation is complex, and designing a controller is challenging. A more straightforward approach is to calculate the system’s state space model. Then, based on this model, we linearize and apply some simplifications to calculate the transfer function of the sway motion based on the velocity commands. Based on Eqs 7, 8, the derivatives of inputs also exist in the sway motion model; we need to select the proper state vector to eliminate the derivatives of the input to calculate the state space. The crane is controlled with velocity commands in the cylindrical coordinate system; we choose them as the input of the system $u_{\mathrm{sys}}={\left[{\dot{\theta }}_{1},\dot{x},\dot{z}\right]}^{T}$ . Now, by selecting the appropriate state vector, the state-space representation of the system is derived as Eq. 10. For more details, see the Appendix B.

$$\begin{array}{cc} x_{1} & =\theta_{5}\\ x_{2} & =\dot{\theta_{5}}-a_{5}{\dot{\theta }}_{1}-a_{6}\dot{x}-a_{7}\dot{z}\\ x_{3} & =\theta_{6}\\ x_{4} & ={\dot{\theta }}_{6}-b_{5}{\dot{\theta }}_{1}-b_{6}\dot{x}-b_{7}\dot{z}\\ x_{5} & =\theta_{1}\\ x_{6} & =x\\ x_{7} & =z \end{array}$$ (10)

Then state space in Eq. 10 linearized around $x_{\mathrm{sys}}={\left[x_{1},x_{2},x_{3},x_{4},x_{5},x_{6},x_{7}\right]}^{T}={\left[0,0,0,0,0,0,3,0\right]}^{T}$ , and based on that the transfer function of the sway motion can be calculated as Eq. 11.

$$\begin{array}{cc} {\dot{\theta }}_{5} & =G_{11}{\dot{\theta }}_{1}+G_{12}\dot{x}+G_{13}\dot{z}\\ {\dot{\theta }}_{6} & =G_{21}{\dot{\theta }}_{1}+G_{22}\dot{x}+G_{23}\dot{z} \end{array}$$ (11)

After calculating the DC gain for $G_{ij}$ , $DC_{G_{12}}=DC_{G_{13}}=DC_{G_{23}}\approx 0$ , so they are negligible, and $G_{11}$ and $G_{22}$ are as follows:

$$\begin{array}{cc} G_{11} & =\frac{\frac{1}{l_{1}+l_{2}}s^{2}}{s^{2}+\frac{c_{1}}{m{\left(l_{1}+l_{2}\right)}^{2}}s+\frac{g}{l_{1}+l_{2}}}\\ G_{22} & =\frac{\frac{3}{l_{2}}s^{2}}{s^{2}+\frac{c_{2}}{ml_{2}^{2}}s+\frac{g}{l_{2}}} \end{array}$$ (12)

Since $l_{1}$ is smaller than $l_{2}$ , the effect of $l_{1}$ is negligible. Furthermore, given that the grapple has a significant weight compared to the first link, we simplify the model by considering $m_{2}=m$ . In this case, the natural frequencies of the two controllers are too close (as we expected), thereby justifying the consideration of the same natural frequency for both motion, perpendicular and parallel motion.

In this paper, the objective of designing the controller is to dampen the sway motions. Therefore, the approach involves considering a controller with zeros precisely at the positions of the poles of the sway motions, as shown in Eq. 13.

$$G_{FF}=\frac{\frac{l_{2}}{g}s^{2}+\frac{c_{2}}{mgl_{2}}s+1}{a_{2}s^{2}+a_{1}s+1}$$ (13)

**FIGURE 3:**
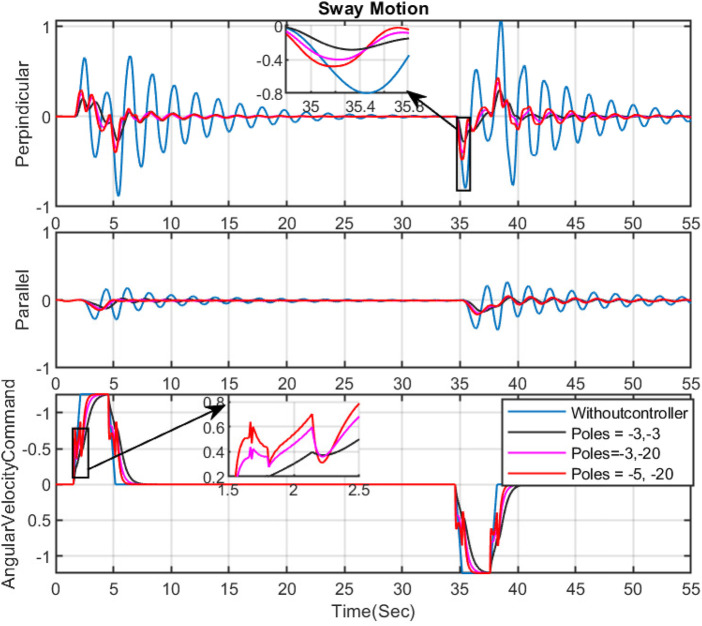
The effect of changing the place of poles on sway motion and input command-Slew motion.

**FIGURE 4:**
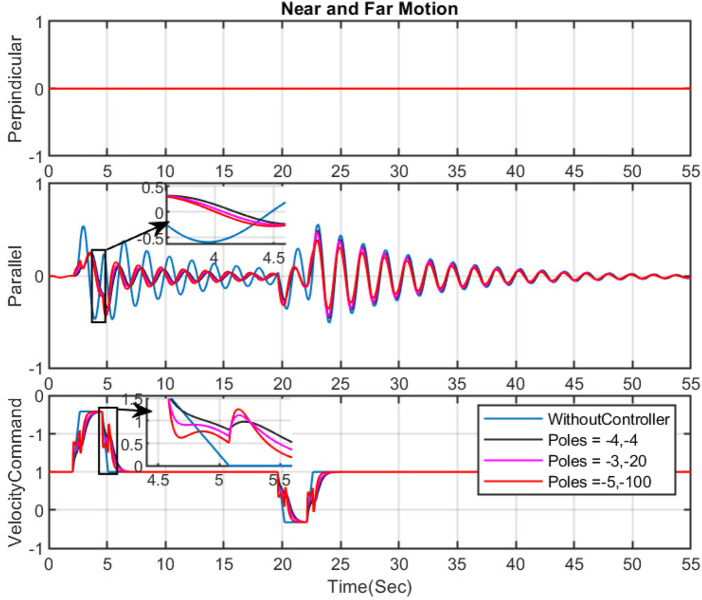
The effect of changing the place of poles on sway motion and input command- Near and Far motion.

The numerator coefficient is calculated based on the identified sway motion model, and if the grapple parameters change, they must be recalculated. The denominator coefficients are tuned based on the controller’s performance. In manual tuning, the person adjusting the controller tunes the parameters based on their experience, intuition, and observations of the system’s performance. This approach heavily relies on the tuner’s expertise and understanding of how different parameters affect the system’s behavior. Furthermore, manual tuning can be more time-consuming and might only sometimes yield the optimal parameter settings. For instance, to assess the impact of pole configurations on sway reduction and the operational speed of the controller, several simulations were conducted by using the simulator, and some example results are presented in Figures 3, 4. In this case, the proposed design approach can be done as follows:

• Choose the initial value for the first pole with the already mentioned rule about five times further from the imaginary axis than sway motions dominant poles.

• Place the second pole slightly less than two times the first pole to ensure smooth shaping of the velocity command

This approach could be used as a starting point. The following section suggests optimal tuning by solving an optimization problem to tune the controller parameters. This method encompasses all criteria necessary to achieve the desired performance of the controller according to customer requirements.

### 3.1 Optimal tuning

This paper proposes an efficient method for determining the optimal coefficients $a_{1}$ and $a_{2}$ , which are crucial for enhancing the performance of the controller design. The approach centers on formulating an optimization problem that directly addresses the controller’s objectives, enabling the boom to move as quickly as possible to improve productivity in loading and unloading logs while ensuring minimal sway motion and maintaining smooth input commands. Customizing the cost function based on specific customer requirements allows for increased flexibility and adaptability in the controller design.

To achieve these goals succinctly, the optimization problem is established to minimize several key terms in the cost function. These terms include the rise time to guarantee speed, the error to ensure the velocity command is accurately followed, and the difference in output to maintain a smooth control command viable for real machine implementation.

Furthermore, the paper delves into the critical considerations for the optimization constraints aimed at maintaining system stability and performance. Constraint (7c) emphasizes the necessity for real poles, selected to prevent the introduction of additional sway motion, thereby allowing for the integration of reactive operator commands. Constraint (7d) further stipulates that these poles be positioned sufficiently far from the imaginary axis to avoid adding extra dynamics to the main system.

By adjusting the optimal coefficients $a_{1}$ and $a_{2}$ and through the strategic formulation of the optimization problem with these considerations, the proposed solution presents a significant advancement in controller design. Manual tuning of these coefficients through trial and error, a process often fraught with frustration and time consumption, is thus efficiently circumvented. Based on these assumptions, the optimization problem can be formulated in Eqs 14a–14e, :

$$\min_{a_{1},a_{2}}\overset{N}{\underset{k=1}{\sum }}L\left(x_{k},u_{k}\right)\;\;$$ (14a)

$$s.t.x_{k+1}=A\left(a_{1},a_{2}\right)x_{k}+B\left(a_{1},a_{2}\right)u_{k}$$ (14b)

$$y_{k}=C\left(a_{1},a_{2}\right)x_{k}+D\left(a_{1},a_{2}\right)u_{k}$$ (14c)

$$a_{1}-2\sqrt{a_{2}}\geq 0$$ (14d)

$$-a_{1}\pm \sqrt{a_{1}^{2}-4a_{2}}\geq 10a_{2}\left(R\left(P_{\mathrm{swaymotions}}\right)\right.$$ (14e)

**FIGURE 5:**
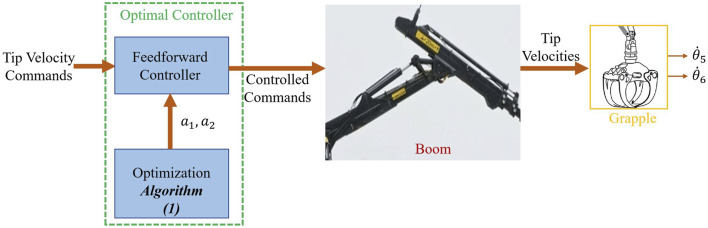
Block diagram for using an optimal feedforward controller in the tip control of a forwarder with less sway motion.

Where $x_{k}$ , $u_{k}$ and $y_{k}$ represent the state, input, and output of blue the feedforward controller $\left(G_{FF}\right)$ at sample time k , respectively. A , B , C , and D are parameter-varying matrices and $a_{1}$ and $a_{2}$ are optimization parameters. $P_{\mathrm{swaymotions}}$ represents the poles of the sway motions, while $R\left(P_{\mathrm{swaymotions}}\right)$ denotes the real part of these sway motion poles. In Eq. 15, the cost function is defined as follows:

$$\begin{array}{cc} L\left(x_{k},u_{k}\right) & =x_{k}^{T}Qx_{k}+\overset{\mathrm{minimize}\;\mathrm{rise}\;\mathrm{time}}{\overbrace{{\left(y_{k}-0.95u_{\mathrm{Max}}\right)}^{T}R\left(y_{k}-0.95u_{\mathrm{Max}}\right)}}\\ & \;+\underset{\mathrm{minimize}\;\mathrm{the}\;\mathrm{error}}{\underbrace{{\left(y_{k}-u_{k}\right)}^{T}S\left(y_{k}-u_{k}\right)}}+\underset{\mathrm{smoothcontrolledcommand}}{\underbrace{\Delta y_{k}^{T}P\Delta y_{k}}} \end{array}$$ (15)

where Q , R , S , and P are weighted positive semi-definite matrices, these can be selected based on the customer’s desired characteristics. In this cost function. Larger value in Q implies a higher penalty for deviations in the corresponding state variables, leading the system to stay close to the desired state, and larger values in R imply a higher penalty for the output being far from this value, thus driving the system to respond faster. Larger values in S imply a higher penalty for this error, promoting the output to track the control input closely, and finally, larger values in P imply a higher penalty for rapid changes in the output, thus ensuring that the control input does not change too abruptly, resulting in smooth control commands. There is a trade-off between the speed of motion and sway damping: moving slowly results in less sway motion. However, it can be frustrating for operators to move the boom slowly, negatively affecting productivity. The customer can choose the weighted matrix based on their preference. The schematic diagram of our proposed methodology is shown in Figure 5. This figure presents a block diagram for employing an optimal feedforward controller in the tip control of a forwarder, designed to reduce sway motion effectively. The discussion and the simulation results of implementing it are depicted in the following section.

## 4 Simulation results and discussion

The implementation of the proposed controller is performed in MATLAB Simulink. The constrained optimization problem is solved by using Casadi (Andersson et al., 2019). It is implemented on the Forwarder Model in Amesim by using interference between Matlab and Amesim. To evaluate the performance of our methodology, we considered two distinct scenarios: one focusing on slew motion and the other on near and far motion. Details are provided in the sections that follow. *Note:* All data presented in the figures have been normalized.

### 4.1 Sway damping in slew motion

**FIGURE 6:**
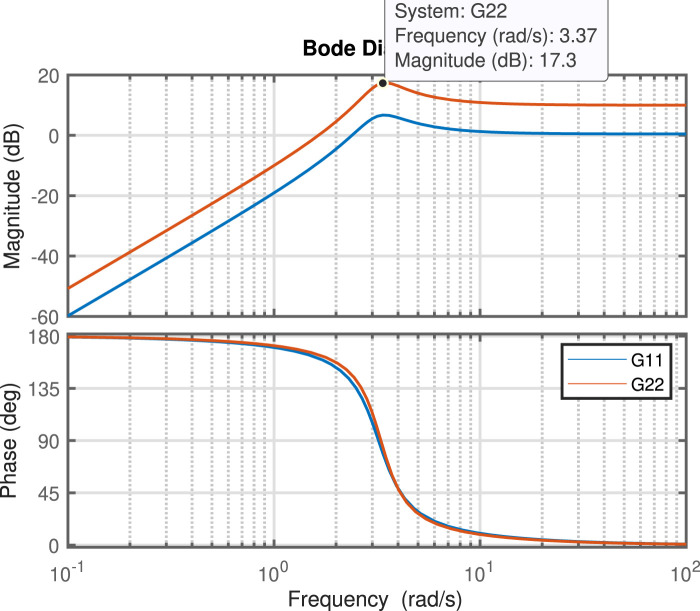
Bode plots for the transfer functions $G_{11}$ and $G_{22}$ .

**FIGURE 7:**
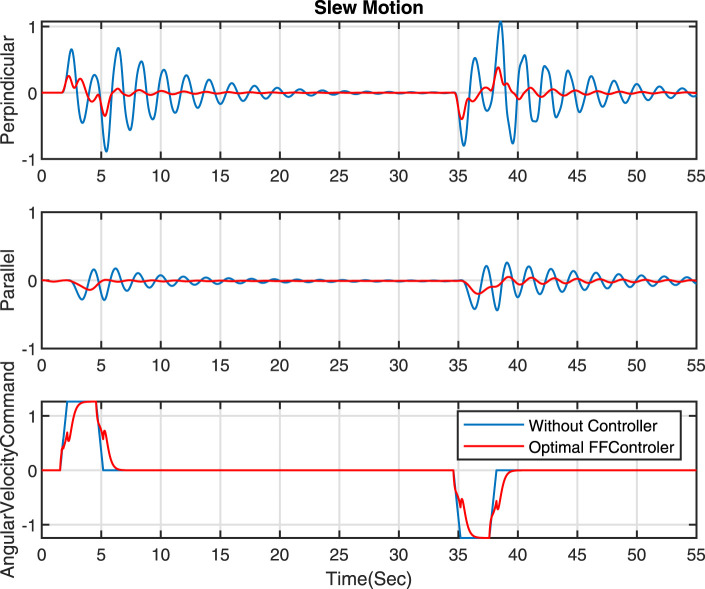
Comparison of optimal FF controller and without a controller - Slew motion.

In this section, our primary objective is to reduce sway motions, especially in perpendicular movements. As mentioned previously, the poles of the controller play a crucial role in enhancing the performance of velocity commands to dampen sway motions. Since the controller designs are based on the natural frequency and nature of the sway motion, before discussing the design of the controller, the Bode plots of $G_{11}$ and $G_{22}$ are presented in Figure 6 to provide a clearer understanding of the natural frequency of the grapple mechanism. To illustrate the impact of different pole positions on the performance of the Feed Forward (FF) controller (Eq. 13) in reducing sway motions, several simulations have been conducted. The results are depicted in Figure 3. This figure demonstrates that selecting poles close to the imaginary axis yields slow and smooth velocity commands, leading to less sway motion. In this figure, the optimal results are achieved with the poles at $p_{\mathrm{controller}}=-3,-3$ compared to others; this results in smoother commands and reduces sway motion. Although the reduction is significant, it results in slower controlled commands. It leads to slower boom movements, potentially extending operation times and adversely affecting productivity, as shown in Figures 3, 4. Conversely, selecting poles far from the imaginary axis results in faster velocity commands. While this reduces sway motion and produces non-smooth commands, it may not be practically implementable on real machines due to potential vibrations at the boom tip during manipulation. Figure 3 shows the importance of solving the optimization problem to calculate the coefficients of the denominator of our Feed forward controller. By solving the optimization problem which is suggested in this paper, the optimal coefficient for the controller in Eq. 13 are calculated as $a_{1}=0.42$ and $a_{2}=0.043$ (equivalently with $p_{\mathrm{controller}}=-5.6307,-4.1253$ ). The Tuning parameters for the cost function are chosen as $Q=I_{2}$ , R=1 , S=3 , and P=1 . The comparison between the scenarios without a controller and using an optimal FF controller is presented in Figure 7. As illustrated, implementing the optimal FF controller (indicated by the red line) significantly reduces sway motion, achieving more than a 63% reduction in the perpendicular direction and over a 67% decrease in the parallel direction during slew movement.

### 4.2 Sway damping in near and far motion

In this section, the near and far movement is studied, and our desired goal is a reduction in the parallel sway motion (in the near and far movement, the sway motion in the perpendicular direction is negligible). Several simulations have demonstrated the effect of various pole positions on the FF controller’s ability to minimize sway motions. The outcomes are presented in Figure 4. Generally, positioning the poles further from the imaginary axis generates faster velocity commands. While this approach decreases sway motion and yields fewer smooth commands, it may not be feasible for the actual machine due to possible vibrations at the boom tip during handling. Figure 4 highlights the significance of addressing the optimization problem to determine the coefficients for the denominator of our feed forward controller.

**FIGURE 8:**
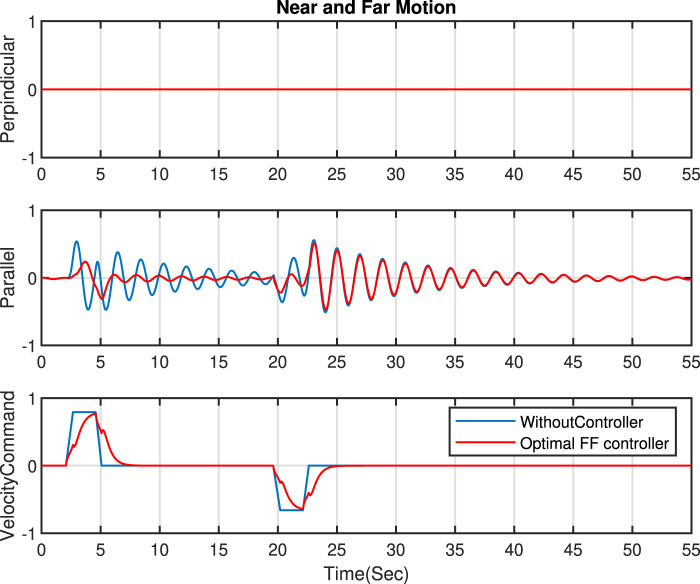
Comparison of optimal FF controller and without controller - Near and Far motion.

Through the resolution of the optimization issue proposed in this study, we obtain the optimal coefficients for the controller in Eq. 7, with $a_{1}=0.708$ and $a_{2}=0.12$ (equivalently with $p_{\mathrm{controller}}=-3.5576,-2.3424$ ). The tuning parameters for the cost function have been set at $Q=I_{2}$ , R=2 , S=0 , and P=0.03 . A comparison between scenarios with and without applying an optimal FF controller is showcased in Figure 8. The results demonstrate that utilizing the optimal FF controller (represented by the red line) markedly decreases sway motion, with a reduction exceeding 63% in the perpendicular direction and more than 67% in the parallel direction during slew movement.

## 5 Conclusion

This study presents a novel feedforward control algorithm for robotic manipulators with passive grappling mechanisms by significantly reducing grapple sway motion. We began with a theoretical analysis of the problem, followed by designing and implementing a feedforward controller based on the nature of the sway motions. This was succeeded by solving the optimization problem to fine-tune the controller’s parameters. The designed method was implemented through co-simulation between AMESim and MATLAB Simulink.

The novel controller is applied for tip control on the forwarder, and the results show a significant reduction in the perpendicular direction (for slew movement) and the parallel direction (near and far movement). The results demonstrate that the optimal FF controller achieves a reduction of over 60% in both directions without additional sway-detection hardware. This simplifies the system’s architecture and cuts costs, making it a practical and cost-effective solution for sway control and instilling confidence in its economic viability.

The controller operates remarkably efficiently, demanding minimal computational resources while maintaining a smooth performance. It effectively dampens sway without compromising its effectiveness, demonstrating robustness and adaptability under initial sway conditions. Its computational efficiency and minimal hardware requirements underscore its potential as an economical and practical solution for sway prevention, instilling confidence in its effectiveness.

Our method’s adaptability and wide-ranging usefulness suggest its potential for widespread adoption in diverse industries, including construction, forestry, and aerial payload transport. It offers a flexible approach to designing anti-sway controls, thereby improving safety and efficiency in operations. The techniques and insights we’ve shared significantly contribute to the field of manipulator control, paving the way for new advancements in stabilizing machinery. Our research underscores how the strategic use of sophisticated control algorithms can enhance performance, safety, and cost-effectiveness in operations, fostering optimism about its potential.

## Data Availability

The data supporting the findings of this study, including the simulated model of the crane, are confidential. As such, they are not publicly available due to commercial restrictions. However, a summary of the data and methodology can be provided upon reasonable request to the corresponding author, subject to confidentiality agreements.

## Appendix A

This appendix provides detailed formulations of the kinetic energy (T) and potential energy (V) used in the analysis of the sway dynamics of the grapple (Eq. 4). Part of the following equations are reproduced from Kalmari et al. (2013) and customized to our case. These formulations are essential for understanding the derivation of the Lagrangian, as referenced in the main body of the manuscript.

To apply the Lagrangian formulation, the kinetic and potential energy of the system are calculated. It is assumed that the entire mass of the system is concentrated at a single point, resulting in no rotational inertia. Considering the inertial tensor would significantly increase the complexity of the dynamic equations. However, in this case, the angular velocities are low, and the rotational inertia is deemed insignificant. In Figure 2B The position of the mass $\left(x_{m},y_{m},z_{m}\right)$ relative to the tip position is calculated as follows:

$$\left\{\begin{array}{c} x_{m}=-l_{2}\sin\left(\theta_{6}\right)\;\\ y_{m}=l_{3}\sin\left(\theta_{5}\right)\;\\ z_{m}=-l_{3}\cos\left(\theta_{5}\right)\; \end{array}\right.$$
(A1)

and accordingly its position in the base frame $\left(x_{t},y_{t},z_{t}\right)$ is calculated as follows:

$$\left\{\begin{array}{c} x_{t}=x_{\mathrm{tip}}+x_{m}\cos\left(\theta_{1}\right)-y_{m}\sin\left(\theta_{1}\right)\;\\ y_{t}=y_{\mathrm{tip}}+x_{m}\sin\left(\theta_{1}\right)+y_{m}\cos\left(\theta_{1}\right)\;\\ z_{t}=z_{\mathrm{tip}}+z_{m}\; \end{array}\right.$$
(A2)

where $\left(x_{\mathrm{tip}},y_{\mathrm{tip}},z_{\mathrm{tip}}\right)$ is position of the tip respect to base frame. we can now calcuate the kinetic energy:

$$\begin{array}{cc} T & =\frac{1}{2}mv^{2}=\frac{1}{2}m\left({\dot{x}}_{t}^{2}+{\dot{y}}_{t}^{2}+{\dot{z}}_{t}^{2}\right)\\ & =0.5m\left({\dot{x}}_{\mathrm{tip}}+l_{2}\sin\left(\theta_{6}\right)\sin\left(\theta_{1}\right){\dot{\theta }}_{1}\right.\\ & \;\left.-l_{3}\cos\left(\theta_{5}\right)\sin\left(\theta_{1}\right){\dot{\theta }}_{5}-l_{3}\sin\left(\theta_{5}\right)\cos\left(\theta_{1}\right){\dot{\theta }}_{1}\right.\\ & \;-l_{2}\cos\left(\theta_{6}\right)\cos\left(\theta_{1}\right){\dot{\theta }}_{6}{\left.+l_{2}\sin\left(\theta_{5}\right)\sin\left(\theta_{6}\right)\sin\left(\theta_{1}\right){\dot{\theta }}_{6}^{2}\right)}^{2}\\ & \;+0.5m\left({\dot{y}}_{\mathrm{tip}}-l_{2}\sin\left(\theta_{6}\right)\cos\left(\theta_{1}\right){\dot{\theta }}_{1}+l_{3}\cos\left(\theta_{5}\right)\cos\left(\theta_{1}\right){\dot{\theta }}_{5}\right.\\ & \;\left.-l_{3}\sin\left(\theta_{5}\right)\sin\left(\theta_{1}\right){\dot{\theta }}_{1}-l_{2}\cos\left(\theta_{6}\right)\sin\left(\theta_{1}\right){\dot{\theta }}_{6}\right.\\ & \;{\left.+l_{2}\sin\left(\theta_{5}\right)\sin\left(\theta_{6}\right)\cos\left(\theta_{1}\right){\dot{\theta }}_{6}^{2}\right)}^{2}\\ & \;+0.5m{\left({\dot{z}}_{\mathrm{tip}}+l_{3}\sin\left(\theta_{5}\right){\dot{\theta }}_{5}+l_{2}\cos\left(\theta_{5}\right)\sin\left(\theta_{6}\right){\dot{\theta }}_{6}^{2}\right)}^{2} \end{array}$$
(A3)

On the other hand, the potential energy is given by:

$$V=mgh=mg\left(z_{\mathrm{tip}}-l_{3}\cos\left(\theta_{5}\right)\right)$$
(A4)

### Appendix B

In this Appendix, detailed derivations and supplementary formulas used throughout the Section 3 are provided as follows: The parameters $a_{i}$ , for i=5,6,7 in Eq. 10 are calculated after simplification as follows:

$$\begin{array}{cc} a_{5}= & -\left(\left(m_{0}l_{0}+ml_{3}\right)cos\left(x_{1}\right)cos\left(x_{5}\right)cos\left(x_{5}\right)x_{7}\right.\\ & +\left.cos\left(x_{1}\right)sin\left(x_{5}\right)sin\left(x_{5}\right)x_{7}\right)\\ & -\left.ml_{3}\left(-l_{3}+l_{2}cos\left(x_{1}\right)sin\left(x_{3}\right)\right)\right)/\left(m\left(l_{0}^{2}+l_{3}^{2}\right)\right)\\ a_{6}= & -\left(\left(m_{0}l_{0}+ml_{3}\right)\left(cos\left(x_{1}\right)cos\left(x_{5}\right)\left(cos\left(x_{5}\right)sin\left(x_{5}\right)\right.\right.\right.\\ & -\left.\left.\left.\left(cos\left(x_{5}\right)cos\left(x_{1}\right)sin\left(x_{5}\right)\right)\right)\right)\right)/\left(\left(m_{0}l_{0}^{2}+ml_{3}^{2}\right)\right)\\ a_{7}= & \;0 \end{array}$$
(B1)

and $b_{i}$ , for i=5,6,7

$$\begin{array}{cc} b_{5}= & -\left(1/l_{2}\right)\left(sin\left(x_{1}\right)\left(l_{2}+l_{1}cos\left(x_{3}\right)\right)\right.\\ & +\left.sin\left(x_{5}\right)x_{7}+cos\left(x_{5}\right)x_{7}\right)\\ b_{6}= & -\left(1/l_{2}\right)\left(sin\left(x_{5}\right)\left(cos\left(x_{3}\right)sin\left(x_{5}\right)\right.\right.\\ & +\left.cos\left(x_{5}\right)sin\left(x_{1}\right)sin\left(x_{3}\right)\right)\\ & -\left.cos\left(x_{5}\right)\left(cos\left(x_{3}\right)cos\left(x_{5}\right)-sin\left(x_{1}\right)sin\left(x_{3}\right)sin\left(x_{5}\right)\right)\right)\\ b_{7}= & -\left(1/l_{2}\right)\left(cos\left(x_{1}\right)sin\left(x_{3}\right)\right) \end{array}$$
(B2)

After linearizing Eq. 10, the state space matrices can be calculated as follows:

$$A_{\mathrm{sys}}=\left(\begin{array}{ccccccc} 0 & 1 & 0 & 0 & 0 & 0 & 0\\ -10.33 & 0 & -0.1966 & 0 & 0 & 0 & 0\\ 0 & 0 & 1 & 0 & 0 & 0 & 0\\ 0 & -10.9 & 0 & -0.1793 & 0 & 0 & 0\\ 0 & 0 & 0 & 0 & 0 & 0 & 0\\ 0 & 0 & 0 & 0 & 0 & 0 & 0\\ 0 & 0 & 0 & 0 & 0 & 0 & 0 \end{array}\right)$$

$$B_{\mathrm{sys}}=\left(\begin{array}{ccc} 0 & 1.053 & 0\\ -5.556 & 0 & 0\\ 0 & 0 & -0.207\\ 0.9959 & 0 & 0\\ 1 & 0 & 0\\ 0 & 1 & 0\\ 0 & 0 & 1 \end{array}\right)$$

$$C_{\mathrm{sys}}=\left[\begin{array}{ccccccc} 0 & 1 & 0 & 0 & 0 & 0 & 0\\ 0 & 0 & 0 & 1 & 0 & 0 & 0 \end{array}\right]$$

$$D_{\mathrm{sys}}=\left[\begin{array}{ccc} 0 & 1.053 & 0\\ -5.556 & 0 & 0 \end{array}\right]$$

## References

[B1] Andersson J. A. Gillis J. Horn G. Rawlings J. B. Diehl M. (2019). Casadi: a software framework for nonlinear optimization and optimal control. Math. Program. Comput. 11, 1–36. 10.1007/s12532-018-0139-4

[B2] Ayoub E. Levesque P. Sharf I. (2023). “Grasp planning with cnn for log-loading forestry machine,” in 2023 IEEE international conference on robotics and automation (ICRA) (IEEE), 11802–11808.

[B3] Brkić V. S. Klarin M. Brkić A. D. (2015). Ergonomic design of crane cabin interior: the path to improved safety. Saf. Sci. 73, 43–51. 10.1016/j.ssci.2014.11.010

[B4] Cole M. O. (2011). A discrete-time approach to impulse-based adaptive input shaping for motion control without residual vibration. Automatica 47, 2504–2510. 10.1016/j.automatica.2011.08.039

[B5] Dvořák J. Malkovskỳ Z. Macků J. (2008). Influence of human factor on the time of work stages of harvesters and crane-equipped forwarders. J. For. Sci. 54, 24–30. 10.17221/790-jfs

[B6] Fielding S. Nahon M. (2019). “Input shaped trajectory generation and controller design for a quadrotor-slung load system,” in 2019 International conference on unmanned aircraft systems (ICUAS) (IEEE), 162–170.

[B7] Hera P. L. Morales D. O. (2015). Model-based development of control systems for forestry cranes. J. Control Sci. Eng. 2015, 27. 10.1155/2015/256951

[B8] Jebellat I. Sharf I. (2023). “Trajectory generation with dynamic programming for end-effector sway damping of forestry machine,” in 2023 IEEE international conference on robotics and automation (ICRA) (IEEE), 8134–8140.

[B9] Kalmari J. Backman J. Visala A. (2014). Nonlinear model predictive control of hydraulic forestry crane with automatic sway damping. Comput. Electron. Agric. 109, 36–45. 10.1016/j.compag.2014.09.006

[B10] Kalmari J. Hyyti H. Visala A. (2013). Sway estimation using inertial measurement units for cranes with a rotating tool. IFAC Proc. Vol. 46, 274–279. 10.3182/20130626-3-au-2035.00050

[B11] Liu Y. Jiang D. Yun J. Sun Y. Li C. Jiang G. (2022). Self-tuning control of manipulator positioning based on fuzzy pid and pso algorithm. Front. Bioeng. Biotechnol. 9, 817723. 10.3389/fbioe.2021.817723 35223822 PMC8873531

[B12] Pai M.-C. (2012). Closed-loop input shaping control of vibration in flexible structures via adaptive sliding mode control. Shock Vib. 19, 221–233. 10.1155/2012/803479

[B13] Qiang H.-y. Sun Y.-g. Lyu J.-c. Dong D.-s. (2021). Anti-sway and positioning adaptive control of a double-pendulum effect crane system with neural network compensation. Front. Robotics AI 8, 639734. 10.3389/frobt.2021.639734 PMC8092389 33954163

[B14] Reis J. Yu G. Cabecinhas D. Silvestre C. (2023). High-performance quadrotor slung load transportation with damped oscillations. Int. J. Robust Nonlinear Control 33, 10227–10256. 10.1002/rnc.6306

[B15] Sadr S. Moosavian S. A. A. Zarafshan P. (2014). Dynamics modeling and control of a quadrotor with swing load. J. Robotics 2014, 1–12. 10.1155/2014/265897

[B16] Solatges T. Rubrecht S. Rognant M. Bidaud P. (2017). “Adaptive input shaper design for flexible robot manipulators,” in 2017 IEEE/RSJ international conference on intelligent robots and systems (IROS) (IEEE), 444–449.

[B17] ur Rehman S. F. Mohamed Z. Husain A. Jaafar H. Shaheed M. Abbasi M. (2022). Input shaping with an adaptive scheme for swing control of an underactuated tower crane under payload hoisting and mass variations. Mech. Syst. Signal Process. 175, 109106. 10.1016/j.ymssp.2022.109106

[B18] Yousefi E. Losey D. P. Sharf I. (2022). “Assisting operators of articulated machinery with optimal planning and goal inference,” in 2022 international conference on robotics and automation (ICRA) (IEEE), 2832–2838.

[B19] Zhou X. Xu Z. Li S. (2019). Collision-free compliance control for redundant manipulators: an optimization case. Front. neurorobotics 13, 50. 10.3389/fnbot.2019.00050 PMC6662470 31396070

