# Corrigendum: Optimal sway motion reduction in forestry cranes

**DOI:** 10.3389/frobt.2024.1491980

**Published:** 2024-10-01

**Authors:** Elham Kowsari, Reza Ghabcheloo

**Affiliations:** Faculty of Engineering and Natural Sciences, Tampere University, Tampere, Finland

**Keywords:** sway damping, optimal control, forestry machinery automation, forwarder, feedforward (FF) control

In the published article, there was an error in Figure 7 as published. The figure was incorrectly replaced with a duplicate of Figure 4, resulting in the intended Figure 7 not being displayed. The corrected Figure 7 and its caption appear below.

**FIGURE 7:**
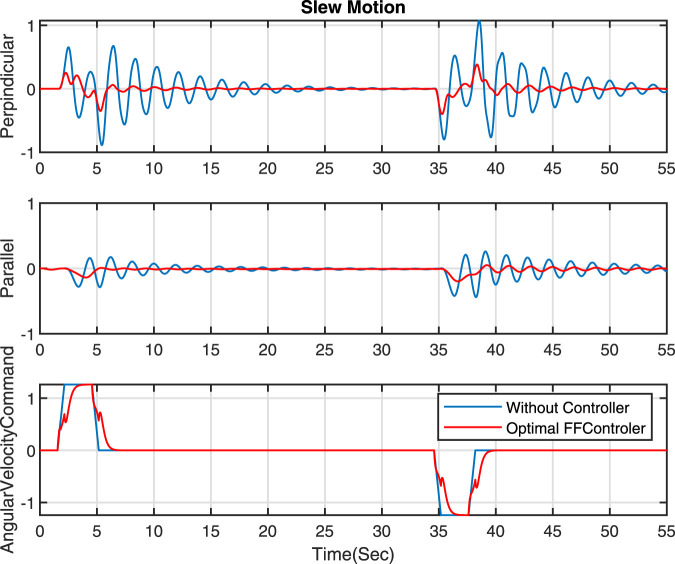
Comparison of optimal FF controller and without a controller - Slew motion.

The authors apologize for this error and state that this does not change the scientific conclusions of the article in any way. The original article has been updated.

